# Intravenous immune globulin in hereditary inclusion body myopathy: a pilot study

**DOI:** 10.1186/1471-2377-7-3

**Published:** 2007-01-29

**Authors:** Susan Sparks, Goran Rakocevic, Galen Joe, Irini Manoli, Joseph Shrader, Michael Harris-Love, Barbara Sonies, Carla Ciccone, Heidi Dorward, Donna Krasnewich, Marjan Huizing, Marinos C Dalakas, William A Gahl

**Affiliations:** 1 Medical Genetics Branch, National Human Genome Research Institute, National Institutes of Health, Bethesda, MD, USA; 2 Department of Genetics and Metabolism, Children's National Medical Center, Washington DC, USA; 3 Neuromuscular Diseases Section, National Institute of Neurological Disorders and Stroke, National Institutes of Health, Bethesda, MD, USA; 4 Department of Rehabilitation Medicine, Clinical Center, National Institutes of Health, Bethesda, MD, USA; 5 George Washington University School of Medicine and Health Sciences, Washington DC, USA; 6 University of Maryland, Department of Hearing and Speech Sciences, College Park, MD, USA

## Abstract

**Background:**

Hereditary Inclusion Body Myopathy (HIBM) is an autosomal recessive, adult onset, non-inflammatory neuromuscular disorder with no effective treatment. The causative gene, *GNE*, codes for UDP-*N*-acetylglucosamine 2-epimerase/*N*-acetylmannosamine kinase, which catalyzes the first two reactions in the synthesis of sialic acid. Reduced sialylation of muscle glycoproteins, such as α-dystroglycan and neural cell adhesion molecule (NCAM), has been reported in HIBM.

**Methods:**

We treated 4 HIBM patients with intravenous immune globulin (IVIG), in order to provide sialic acid, because IgG contains 8 μmol of sialic acid/g. IVIG was infused as a loading dose of 1 g/kg on two consecutive days followed by 3 doses of 400 mg/kg at weekly intervals.

**Results:**

For all four patients, mean quadriceps strength improved from 19.0 kg at baseline to 23.2 kg (+22%) directly after IVIG loading to 25.6 kg (+35%) at the end of the study. Mean shoulder strength improved from 4.1 kg at baseline to 5.9 kg (+44%) directly after IVIG loading to 6.0 kg (+46%) at the end of the study. The composite improvement for 8 other muscle groups was 5% after the initial loading and 19% by the end of the study. Esophageal motility and lingual strength improved in the patients with abnormal barium swallows. Objective measures of functional improvement gave variable results, but the patients experienced improvements in daily activities that they considered clinically significant. Immunohistochemical staining and immunoblotting of muscle biopsies for α-dystroglycan and NCAM did not provide consistent evidence for increased sialylation after IVIG treatment. Side effects were limited to transient headaches and vomiting.

**Conclusion:**

The mild benefits in muscle strength experienced by HIBM patients after IVIG treatment may be related to the provision of sialic acid supplied by IVIG. Other sources of sialic acid are being explored as treatment options for HIBM.

## Background

Hereditary inclusion body myopathy (HIBM; OMIM 600737) is an adult-onset autosomal recessive myopathy that typically presents with distal muscle weakness, usually foot drop, in the second or third decade of life [1-3]. The disease progresses to require wheelchair confinement in two to three decades. The quadriceps muscles are relatively spared, at least clinically, even late in the disease. Muscle histology shows vacuolated fibers, variation of fiber size, increased connective tissue, and fibrofatty replacement of muscle fibers. Inflammation is typically absent.

HIBM is rare, but a Persian Jewish isolate [4] enabled investigators to map the condition to chromosome 9p12–13. The causative gene, *GNE*, was subsequently identified and a common mutation, M712T, was found among affected Persian Jews [5]. The *GNE *gene encodes the bifunctional enzyme, UDP-*N*-acetylglucosamine (UDP-GlcNAc) 2-epimerase/*N*-acetylmannosamine (ManNAc) kinase, catalyzing the rate-limiting steps in sialic acid synthesis [6,7]. The M712T mutation is located within the kinase domain of the *GNE *gene. Nearly 20 other *GNE *mutations have been described in HIBM patients of different ethnic backgrounds [8-12]; a condition allelic to HIBM, Distal Myopathy with Rimmed Vacuoles (DMRV), occurs in Japan [13-15]. HIBM-associated *GNE *mutations, whether in the epimerase or the kinase domain, result in reduced activity of both UDP-GlcNAc 2-epimerase and ManNAc kinase [16,17]. These decrements are considered responsible for reduced production of sialic acid, a negatively charged sugar that serves as the terminal carbohydrate on glycoconjugates [18]. Glycosylation of proteins is critical for proper folding of nascent proteins, resistance to proteases, intracellular trafficking, and cell-cell interactions [19,20].

The pathogenic mechanism of muscle fiber degeneration in HIBM remains unknown. However, evidence suggests that decreased availability of sialic acid causes hyposialylation of muscle glycoproteins, whether involving glycans in general [17,21], O-linked glycans [22], polysialic acid on neural cell adhesion molecules (PSA-NCAM) [23], or specific O-mannosylated glycosyl residues on α-dystroglycan [24]. These latter oligosaccharides govern interactions of α-dystroglycan with extracellular matrix proteins [25,26], and their deficiency is responsible for several congenital muscular dystrophies, including Walker-Warburg syndrome, Muscle-Eye-Brain disease, and Fukuyama's muscular dystrophy [27-29]. In HIBM, decreased sialylation of α-dystroglycan could impair interaction with other skeletal muscle proteins essential for function.

We reasoned that provision of sialic acid to HIBM patients could help normalize the sialylation status of muscle glycoproteins and provide clinical benefit. Because no approved source of free sialic acid is available, we delivered this charged sugar via intravenous immune globulin G (IVIG), a glycoprotein that contains 8 μmoles of sialic acid per gram [30]. Maintaining high levels of serum IgG could result in continuous breakdown of the glycoprotein by neuraminidase, providing sufficient quantities of free sialic acid to the blood, scavenger cells and eventually muscle tissue. Side effects included headaches, nausea and vomiting, particularly after the second day of the loading dose; these resolved within 48 hours. Improvements in quantitative muscle testing and the six-minute walk were recorded. Qualitative improvements in activities of daily living, muscle strength, and endurance were noted in all four treated patients.

## Methods

### Protocol design

Four patients with molecularly proven HIBM were enrolled in a protocol approved by the NHGRI Institutional Review Board, and gave written, informed consent. Baseline evaluations at the NIH Clinical Center consisted of a complete history and physical examination, laboratory studies including quantitative immune globulins, a cardiac evaluation with a chest radiograph, electrocardiogram and echocardiogram, an ophthalmologic examination, and a neuromuscular evaluation that included an MRI of the calf and/or thigh. A baseline Rehabilitation Medicine evaluation included quantitative muscle strength testing, i.e., isometric fixed dynamometry [31] of 10 muscle groups, and measurements of tongue strength [32,33]. Strength was tested with subjects positioned supine and sitting on a treatment table, with stabilization provided by belts or examiners. Subjects performed two maximal voluntary isometric contractions of 10 muscle groups bilaterally separated by at least 30 s of rest. Joint angles were kept constant throughout the study via goniometric measurements. Cuffs were placed around the desired limb and were attached to a force transducer fixed to a stationary bed frame. The means of two trials were used for data analyses. Functional testing included a modified barium swallow evaluation, functional reach tests [34], timed up and go [34], and a 6-minute walk test to measure endurance [35]. A quadriceps muscle biopsy was taken for histology, immunohistochemistry and immunoblotting. Pulmonary function testing was performed in patients 3 and 4.

After baseline testing, patients received a loading dose (1 g/kg) of immune globulin (Octagam 5% or Gamunex 10%) by continuous intravenous infusion on two consecutive days. Patients were hydrated before and after the infusions with 500 mL of either normal saline or half-normal saline. They were also premedicated with 50 mg of IV diphenhydramine and either 650 mg of acetaminophen or 250 mg of naproxen. One week after the two loading doses, the patients began receiving weekly maintenance doses of IVIG at 400 mg/kg for a total of 3 maintenance infusions. Hydration and premedication were also provided for these infusions. Evaluations of quantitative muscle testing, grip and pinch strength, 6-minute walk test and functional reach studies were performed directly after the loading dose and at the end of the study. Tongue strength measurements, a modified barium swallow, an ophthalmologic evaluation and a muscle biopsy were repeated at the end of the study. Laboratory testing was performed after each infusion and at the end of the study to monitor for safety and to follow levels of immune globulins.

### Muscle biopsies

Open quadriceps muscle biopsies were performed before and 3–4 days after the last dose of IVIG. Specimens were processed for muscle enzyme histochemistry, immunocytochemistry, and immunoblotting as described below.

### Alpha-dystroglycan and NCAM immunocytochemistry

Coded serial cryosections (5 μm each), obtained before and after IVIG treatment from the patients, one normal control (N_1_), and one individual with sporadic IBM (N_2_), were fixed in acetone for 5 min and pre-incubated with phosphate-buffered saline (PBS) containing 2% bovine serum albumin (BSA). The sections were then incubated overnight at 4°C with antibodies against α-dystroglycan (VIA4-1 and IIH6, Upstate, Lake Placid, NY, USA) diluted at 1:100, or anti-NCAM (123C3, Santa Cruz Biotechnologies, CA, USA) diluted at 1:200. Immunoreactivity was detected using FITC-labeled goat anti-mouse IgG or IgM (Molecular Probes, Eugene, OR) at a dilution of 1:500 for 1 h. Negative controls included the omission of each primary antibody in every staining. The sections were mounted in 30% glycerol-based medium and viewed using fluorescent microscopy.

### Immunoblotting

Muscle tissues were homogenized in 200 μL of CelLytic (CelLytic, Sigma, St Louis, MO, USA), consisting of a mild detergent, bicine buffer and 150 mM NaCl, with protease inhibitors (Complete Mini^®^, Roche, Mannheim, Germany). The lysates were sonicated and microfuged at 8000 g for 10 min at 4°C. The protein concentration of the supernatant was determined using the BCA protein assay (Pierce, Rockford, IL, USA) and equal amounts of protein (25–50 μg) were loaded and electrophoresed on 4–12% Tris-Glycine gels (Novex, Invitrogen, Carlsbad, CA, USA). Resolved proteins were electrophoretically transferred to a 0.45 μm Hybond ECL nitrocellulose membrane (Amersham Pharmacia Biotech, IL, USA). Subsequently, membranes were blocked with 10% fat-free milk in PBS with 0.1% Tween 20 (PBST) for 30 min and then incubated with antibodies against NCAM (123C3, Santa Cruz Biotechnologies, CA, USA), α-dystroglycan (clone IIH6C4, 05–593, Upstate, Lake Placid, NY, USA) or β-actin (AAN01, Cytoskeleton, Denver, CO, USA) followed by horseradish peroxidase-conjugated secondary antibodies in PBST containing 3% fat-free milk. Immunoreactive bands were visualized by enhanced chemiluminescence using the Amersham ECL Western Blotting Detection Reagent (GE Healthcare, Buckinghamshire, UK). Density analysis was performed on digital images obtained with the Kodak Image station and software (Perkin Elmer, Boston, MA, USA). The protein levels were normalized to those of β-actin to correct for differences in protein loading and/or transfer.

For enzyme treatment experiments, protein samples (25 μg) were incubated with neuraminidase (N-6514, Sigma, St Louis, MO, USA), 1 mU/μg of protein, for 30 min at 37°C. Undigested and digested samples were analyzed by immunoblotting.

### Isoelectric focusing

For isoelectric focusing (IEF) of transferrin, whole serum was iron-saturated and isofocused on pH4-6.5 PhastGels (Amersham Biosciences, Piscataway, NJ, USA) as described [36,37]. Transferrin isoforms were visualized by incubation with rabbit anti-human transferrin antibody (Dakocytomation, Denmark) followed by silver staining (Amersham Biosciences, Piscataway, NJ, USA). For IEF of apolipoprotein C-III (Apo C-III), whole serum proteins were acetone-delipidated and resuspended in 8 M urea. Subsequently, Apo C-III was isolated and concentrated by centrifugation through centricon filters (Millipore, Bedford, MA, USA) and isofocused on PhastGels pH4-6.5 (Amersham Biosciences, Piscataway, NJ, USA). The proteins were transferred by diffusion blotting onto nitrocellulose membranes and visualized by incubation with goat anti-Apo CIII antibodies (Chemicon, Temecula, CA, USA) and Enhanced ChemiLuminescence detection (Amersham Biosciences, Piscataway, NJ, USA) as described [37,38].

## Results

### Patients

Patient 1 is a 29-year-old Iranian-Jewish woman diagnosed at age 24 years. She presented at age 17 with inability to abduct her legs against any resistance on an exercise machine; other muscle groups were strong. Following her first pregnancy at age 20, she noticed weakness, difficulty climbing stairs, and tripping and falling that progressed in frequency over the next 3 years from once a month to once a week. A muscle biopsy and genetic testing confirmed the diagnosis of HIBM with homozygous M712T mutations in *GNE*. At age 25, she noted difficulty fastening snaps and could not raise her arms to pull back her hair. Progression was gradual, with more significant decline following illnesses. Treatments included solumederol, 1 g intravenously for 3 days, which resulted in improved strength but was followed by relapse. On one occasion, IVIG (dose unknown) was given over 3 days, providing no improvement but complicated by aseptic meningitis requiring hospitalization. For the 6 months prior to admission, the patient used a wheelchair and required assistance walking and with transfers. Medications included sertraline for depression and anxiety, and vitamins.

At the NIH Clinical Center, the patient was wheelchair dependent, but could go from sit to stand and ambulate a few feet with support. She had bilateral foot drop and significant weakness of upper and lower extremities, with some sparing of the quadriceps. Muscle atrophy was universally apparent. Cranial nerves and sensation were intact. MRI of the calf showed pronounced fatty involution of the musculature bilaterally, worse proximally. The lateral soleus and posterior tibial muscles were somewhat spared distally. A quadriceps muscle biopsy revealed moderate type II fiber predominance. The muscles were atrophic (type II worse than type I) and demonstrated necrosis, active degeneration, and red rimmed vacuoles, consistent with severe chronic myopathy without inflammation. Imunohistochemical staining for NCAM, a surface glycoprotein reflecting muscle regeneration [23,39], showed a significant number of positive fibers.

Patient 2 is the 42-year-old brother of patient 1. He was diagnosed with HIBM at 37 years of age, following the diagnosis of his sister. Weakness upon raising his arms overhead first appeared at age 27. At 34, he began tripping and, in the subsequent year, had difficulty climbing stairs and lifting his son. The upper extremities were most affected, but leg weakness was evident by decreased endurance, speed of walking, and getting in and out of a car. He occasionally used his hands to lift his legs. Symptoms were largely stable, but exacerbated by emotional events. He did not receive any treatment for his myopathy, and medications included only multivitamins.

At the NIH Clinical Center, there was wasting of the triceps and the interosseus and thenar muscles of the hand. Contractures of approximately 5–10 degrees were present in the elbows, with decreased range of motion upon supination of the forearm. The patient had no foot drop. Muscles of the shoulder girdle, pinch, grip, and proximal lower extremities exhibited weakness. The gait was normal, and cranial nerves and sensation were intact. MRI of the thigh revealed advanced atrophy with fatty involution in the proximal posterior compartment and adductor magnus muscles bilaterally. The sartorius and rectus femoris muscles were also involved. There was relative sparing of the quadriceps with the exception of the rectus femoris muscles. On biopsy, the quadriceps muscle showed several small vacuolated fibers with a few degenerating fibers, but no inflammation. Immunohistochemical staining was negative for NCAM, indicating negligible damage to individual muscle fibers.

Patient 3 is a 39-year-old woman of Bohemian/Czech/French Canadian ancestry diagnosed with HIBM at the age of 31 years. Following a year of progressive tripping and falling, she awoke one day with numbness from the waist down that spread to her entire body. She was diagnosed with mild multiple sclerosis, based on the presence of oligoclonal bands in her cerebrospinal fluid and a brain MRI showing a possible "spot." Muscle biopsy also provided evidence of inclusion body myopathy. Currently, she has difficulty arising from a supine position due to weak abdominal muscles, weakness in her hands, arms, and shoulders, and inability to climb stairs. She wears ankle braces to walk. She uses bronchodilators for asthma and sertraline for depression. Confirmatory mutational analysis demonstrated compound heterozygous *GNE *mutations, i.e., R246Q and A631V, affecting the epimerase and kinase domains, respectively.

At the NIH Clinical Center, the patient had bilateral foot drop with a compensated gait. She was able to transfer independently from sit to stand and from stand to sit. There was proximal muscle and neck weakness and limited endurance. Cranial nerves and sensation were intact. MRI of the calf showed end-stage myopathy with advanced fatty involution, but with preservation of muscle volume. Signal alteration was present in areas of non-atrophic muscle. The popliteal muscles in the upper calves and the distal posterior tibial muscles were relatively spared. On biopsy, the quadriceps muscle showed significant necrosis with replacement of muscle by fat and connective tissue, but without inflammation. NCAM staining was minimal.

Patient 4 is the 46-year-old brother of patient 3. He was diagnosed with HIBM at the age of 33 years following a one-year history of progressive tripping and falling, decreased muscle strength and inability to lift his foot. As a cabinetmaker, he relied on this action to support the cabinets as he made them. A muscle biopsy was initially read as consistent with either polymyositis or inclusion body myopathy, but intravenous steroids provided no relief, and HIBM was diagnosed. Plateaus were interspersed with declines, which occurred with lack of exercise but not with illnesses. The lower extremity weakness extended throughout the body. The patient currently wears ankle braces to walk, uses an electric wheelchair for distances, and has assistive devices to drive. He has difficulty holding a pen. Medications include acyclovir, Metamucil, multivitamins, tramadol and diazepam.

At the NIH Clinical Center, there was muscle wasting of the triceps and the interosseus muscles of the hands, along with tightness of the heel cords, hips, and hamstrings. The patient was partially independent when going from sit to stand, and when executing transfers to and from his wheelchair. He could ambulate a few steps using ankle-foot orthotics and support from the wall. He had significant weakness in the proximal upper extremities, triceps, wrist and hand, and decreased strength on hip flexion, hip extension, ankle dorsiflexion, and plantar flexion. Deep tendon reflexes could not be elicited in the upper or lower extremities. Sensation and cranial nerve functions were intact. MRI of the calf showed end-stage myopathy with advanced fatty involution but preservation of muscle contours. Signal alteration occurred in areas of the remaining muscle. There was relative sparing of the popliteus, the tibialis posterior, and the flexor digitorum longus muscles. Quadriceps muscle histology revealed vacuolated and moth-eaten muscle fibers, with degeneration and regeneration. Connective tissue was slightly increased with no signs of inflammation. Immunohistochemical staining with NCAM was negative.

### Effects of IVIG on serum IgG

**Figure 1:**
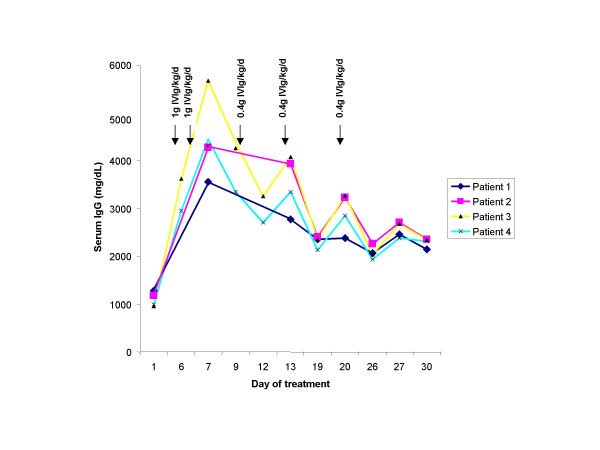
**Serum IgG levels in patients during the course of treatment with IVIG**. Arrows indicate loading doses (1 g/kg) and maintenance doses (0.4 g/kg).

IgG levels during the course of the study are shown in Figure 1. The loading doses of IVIG brought the serum IgG levels to approximately 4 times the baseline, but this concentration of IgG was not sustained by the weekly infusions of 400 mg/kg of IVIG. Throughout the course of the infusion, the patients' serum IgG concentrations ranged from 2 to 4 times the average normal level.

### Muscle strength

**Figure 2:**
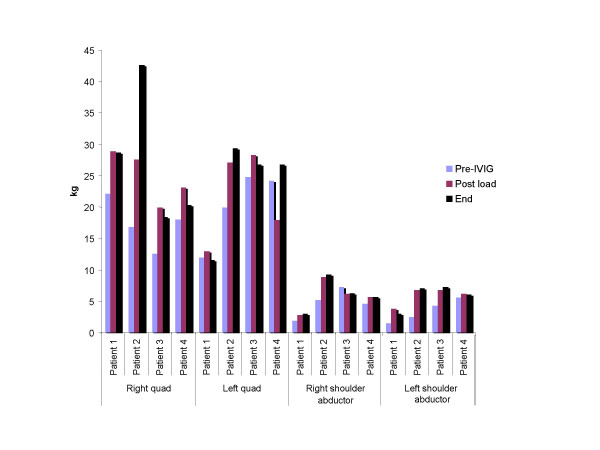
**Quadriceps and shoulder abduction testing in four patients with HIBM**. On average, quadriceps strength increased 22% after IVIG loading and 35% at the end of the study. Shoulder abduction strength increased 44% after IVIG loading and 46% at the end of the study.

On quantitative muscle testing, both distal and proximal muscle groups showed improvement after IVIG treatment. The greatest absolute improvement was in quadriceps strength, as reflected in the capacity to perform work in knee extension. Considering both sides of all 4 patients, mean quadriceps strength improved from 19.0 kg at baseline, to 23.2 kg (+22%) directly after IVIG loading, to 25.6 kg (+35%) at the end of the study. Considered individually, the 4 patients exhibited a 13–150% improvement in the dominant right leg after IVIG treatment (Figure 2). The change in left leg quadriceps strength varied from -3% to +48%. Shoulder abduction also showed significant improvement. Considering both sides of all 4 patients, mean shoulder strength improved from 4.1 kg at baseline, to 5.9 kg (+44%) directly after IVIG loading, to 6.0 kg (+46%) at the end of the study. Considering each patient individually, shoulder abduction on the right side improved 24–79% in three patients and fell 14% in patient 3 (Figure 2). On the left side, shoulder abduction improved 13–184%.

Eight other muscle pairs (right and left), associated with hip flexion, ankle dorsiflexion, elbow flexion and extension, wrist flexion and extension, grip, and pinch, were also evaluated. These muscle groups exhibited variable differences in strength before and after IVIG treatment (Table 2), but the preponderance showed improvement. In fact, on average, the composite improvement for these 8 muscle groups was 5% after the initial IVIG loading and 19% by the end of the study. Tongue strength, which is not known to be affected in HIBM, improved ~5%, from a mean of 63.1 kp at baseline to a mean of 66.2 kp at the end of the study.

### Functional testing

Functional studies included functional reach, timed up and go, and the 6-min walk test. Patient 1 could not perform any of these tests. Patient 4 could not perform the 6-min walk test.

In patient 2, the functional reach after IVIG treatment increased from 7.3 in to 10.2 in (40%) on the right and from 6.0 in to 8.8 in (47%) on the left. However, the functional reach declined in patients 3 and 4 by 8% and 19%, respectively. The time it took to rise from a chair (timed up and go) improved 1.94 s (27%) for patient 2 and 0.81 s (8%) for patient 3; it worsened by 0.84 s (5%) in patient 4. Endurance, as measured by the 6-min walk test, improved after IVIG treatment from 1633 ft to 1744 ft (7%) in patient 2 (normal mean for age and size, 1997 ft) and from 983 ft to 1065 ft (8%) in patient 3 (normal mean, 1637 ft).

Improvements in the two patients with abnormal modified barium swallows were also noted. Patient 1 had normal esophageal peristalsis with only mild delays after IVIG and patient 3 had no reflux or pooling with only delayed initiation after the treatment.

Patients displayed qualitative or subjective improvements as well. Patient 1 claimed improved strength, energy, and balance and needed less support to walk. She could get in and out of a bed or chair more easily. Brushing her hair was easier. She was able to open a bottle of water and cut a bagel without help. After IVIG treatment, she could snap her fingers and produce noise, which she had been unable to do for years. Patient 2 noted improvements in getting out of bed or a car. He could easily wash his hands and face, which was a struggle before treatment. He could reach further and could lift his children; he had more energy to play with them. Both he and his wife noticed that he walked faster. Patient 3 noted improvements with getting out of bed and up from a sitting position. She needed less support to accomplish her activities of daily living and appreciated increased energy. Patient 4 stated that he felt better, but was unable to provide specific examples. The subjective improvements lasted approximately three weeks, after which each of the patients noted a decline in function.

### Serum glycoproteins

Serum transferrin contains four terminal sialic acid residues on its N-linked oligosaccharides [36,37]. On isoelectric focusing, all four oligosaccharides were sialylated in all four patients before IVIG treatment, just after loading, and at the end of the treatment period [see Additional file 1]. Similarly, the O-linked serum glycoprotein Apo C-III [37,38] exhibited a normal contingent of sialylation in all four patients before and after IVIG treatment [see Additional file 1].

### Muscle studies

**Figure 3:**
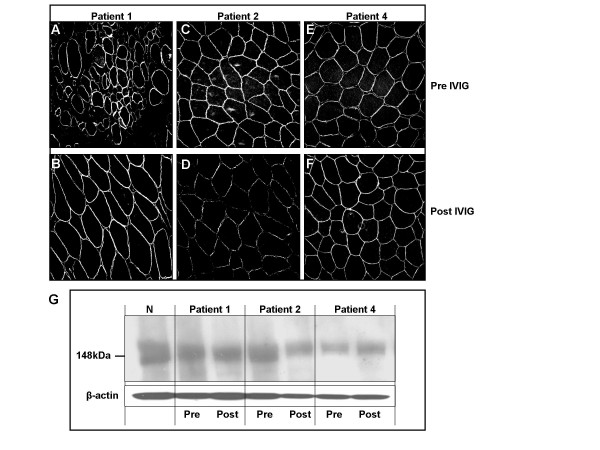
**Expression of α-dystroglycan in quadriceps muscle from HIBM patients**. **A-F**. Immunohistochemistry using antibodies to IIH6. No consistent difference in staining was apparent before (A,C,E) compared with after (B,D,F) IVIG treatment. The specimen shown in A was sampled from a deteriorating area. **G**. Immunoblots of muscle from a normal individual (N) and HIBM patients 1, 2, and 4, labeled with antibodies to α-dystroglycan. No consistent difference in IIH6 staining (reflecting the sialylation status of α-dystroglycan) was apparent before (Pre) compared with after (Post) IVIG treatment. β-Actin bands provide an indication of the level of protein loading.

Muscle MRI examinations were not repeated after IVIG treatment, because one month was not deemed a sufficient amount of time to achieve visible improvement. Standard histology of muscle biopsies showed no difference before compared with after IVIG treatment. The post-treatment biopsy of patient #3 was unsuitable for interpretation, but muscle biopsies from patients 1, 2, and 4 before and after IVIG treatment were available for staining with anti-α-dystroglycan antibodies (VIA4I and IIH6). No consistent differences were seen before and after treatment (Figure 3A–F). Immunoblotting showed no consistent increase in the glycosylation status of α-dystroglycan (using the IIH6 antibody) after IVIG treatment (Figure 3G).

**Figure 4:**
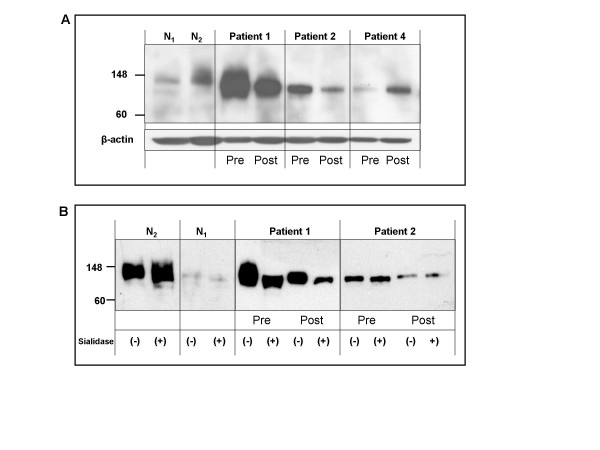
**Expression of sialylated NCAM in muscle of HIBM patients**. **A**. Muscle from two control individuals exhibit NCAM mobility consistent with a higher molecular weight, indicative of sialylation. N_1 _is normal and N_2 _has sporadic IBM; note increased NCAM in this person's muscle. The muscle of patient 1 also has an increased amount of NCAM; patients 2 and 4 have moderate amounts of NCAM. The NCAM of the HIBM patients migrates with greater mobility than normal, indicating lack of sialylation. The amount of muscle NCAM decreased after (Post) compared with before (Pre) IVIG treatment in patients 1 and 2, but increased in patient 4. **B**. Sialidase treatment increased the mobility of control muscle (N_1 _and N_2_). The mobility of muscle NCAM was not altered by sialidase treatment in patient 2, but it was increased by sialidase treatment in patient 1.

NCAM is a sialylated glycoprotein that plays a significant role in muscle fiber development, innervation and regeneration, and whose abundance on the surface of muscle cells reflects the extent of muscle denervation or damage [23,39-42]. Patient 1 exhibited the greatest amount of muscle NCAM before IVIG (Figure 4A), i.e., 4-fold that of the N_1 _(normal) control, relative to actin, as determined by densitometry. After IVIG treatment, the muscle NCAM of patient 1 decreased by 34%. The amount of NCAM decreased by 28% in patient 2, but increased by 58% in patient 4, relative to actin after IVIG administration. In all cases, the HIBM patients' NCAM bands ran lower than the control, reflecting a decreased sialic acid content of this glycoprotein. This was verified for patient 2 by using sialidase to remove terminal sialic acid residues (Figure 4B). Sialidase treatment resulted in greater mobility of the normal muscle NCAM protein, but not that of patient 2 (Figure 4B). Sialidase treatment did, however, reduce the apparent size of muscle NCAM in patient 1, indicating the presence of some sialylation.

NCAM immunohistochemical staining of HIBM muscle (patient 1, 2, and 4) after IVIG treatment did not reveal any consistent differences compared with pre-treatment staining (data not shown).

### Side effects

All patients experienced fatigue and headache following the second loading dose of 1 g/kg IVIG. Patients 1, 2, and 3 had several episodes of nausea and vomiting; patient 2 said it resembled having influenza. Patient 4 had headache and neck stiffness. Patients 1 and 2 experienced anorexia, and patient 2 had abdominal distension and constipation. The headaches were treated with naproxen or acetaminophen. Patient 3 required zofran for relief of the nausea and vomiting. All symptoms resolved by 72 h and did not recur with subsequent maintenance infusions of IVIG. A urinary tract infection was diagnosed incidentally in patient 1, and was treated. The follow-up ophthalmologic evaluation in patient 4 revealed a cottonwool spot near the left optic disc.

Patients 3 and 4 responded to the IVIG with increased sedimentation rates, as high as 96 mm/h (Table 1). Liver enzymes rose slightly in patients 1, 2 and 4; the highest levels were obtained in patient 2, with an ALT of 81 U/L and an AST of 68 U/L. These values subsequently returned to normal. Serum cholesterol levels generally fell, from a mean of 200 mg/dL at baseline to 147 mg/dL just after loading and 173 mg/dL at the end of the study. Remarkably, the four patients excreted 6.4 to 30.8 g/d of glucose in their urine just after the IVIG load, compared with ≤0.1 g/day before and after the study. There were no significant changes in serum electrolytes, glucose, calcium, phosphorus, or bilirubin.

## Discussion

Homozygous or compound heterozygous mutations in either the epimerase or kinase domain of *GNE *cause decreased UDP-GlcNAc 2-epimerase and ManNAc kinase activities in the cells of HIBM patients [16,17]. However, the pathogenic mechanisms responsible for the myopathy of HIBM have not been determined. Measurements of the sialic acid content of glycoproteins have given variable results [17,21-24,43-45]. Using sialylated α-dystroglycan-specific antibodies, Huizing *et al*. first reported decreased muscle α-dystroglycan sialylation in four HIBM patients, each having one mutation in the epimerase domain and one in the kinase domain of *GNE *[24]. Noguchi *et al*. followed with findings of fiber-to-fiber variability in the hyposialylation of α-dystroglycan in the muscles of patients with DMRV, allelic with HIBM [17]. Saito *et al*. reported hyposialylation of muscle glycoproteins in a single patient with DMRV [21], but Hinderlich *et al*. and Salama *et al*. showed no decrease in sialylation of proteins in myoblasts cultured from HIBM patients homozygous for the Persian Jewish *GNE *mutation, *i.e*., M712T [43,44]. Broccolini *et al*. recently reported hyposialylated muscle α-dystroglycan in 4 of 5 HIBM patients, with no effect on binding to laminin [45]; they surmised that the sialylation status of α-dystroglycan is not critical to the protein's function. Ricci *et al*. used NCAM as an indicator of the extent of muscle glycoprotein sialylation; in HIBM, muscle NCAM was found to be hyposialylated [23]. The divergence of these findings allows for the possibility that sialylation abnormalities represent epiphenomena in HIBM, and *GNE *mutations could cause disease by affecting cellular functions not influenced by sialylation.

Indeed, while we verified the hyposialylation of muscle NCAM in our HIBM patients (Figure 4A,B), we found that decreased sialylation of glycoproteins is not a constant finding in HIBM. Isoelectric focusing studies revealed normal N-linked glycosylation of serum transferrin and O-linked glycosylation of Apo C-III [see Addional file 1]. In addition, sialylated α-dystroglycan was not decreased in the muscle of our four HIBM patients prior to IVIG administration. This could be because patients vary with respect to their specific *GNE *mutations, the extent of their sialic acid deficiency, and the degree to which their muscle proteins are sialylated, even when sampling the same muscle. Conditions of the immunohistochemical staining could differ, as could the specificity and sensitivity of the antibodies.

With normal baseline glycosylation of serum glycoproteins and muscle α-dystroglycan in HIBM, we had no opportunity to demonstrate improvement in these parameters after IVIG treatment. Muscle NCAM was hyposialylated before IVIG administration, but we could not discern consistent improvements in NCAM sialylation, either by immunohistochemistry or by immunoblotting, after IVIG treatment. One possible explanation is that the amount of sialic acid provided by IVIG was inadequate to increase glycoprotein sialylation. We estimate that normal adult urinary free sialic acid, a reasonable measure of daily production, approximates 0.3 mmol [46]. Our IVIG loading doses (2 g/kg) should provide 8 μmol/g or ~1 mmol of sialic acid, roughly 3 days' worth of normal sialic acid production. This may or may not be sufficient to achieve a detectable or functional increase in the glycosylation of muscle protein.

We would expect a decrease in muscle NCAM to accompany the decrease in muscle degeneration hoped for with IVIG treatment, and this occurred in patients 1 and 2. The increased amount of hyposialylated NCAM in patient 4 after IVIG treatment contradicts this finding and reflects variability either in response to treatment or in muscle sampling.

In contrast to the absence of consistent effects on histological and immunological markers of sialylation, IVIG treatment improved objective and subjective measures of muscle strength and function in HIBM patients. Muscles that had greater strength at baseline, such as the quadriceps, appeared to benefit more than muscles that were already extensively damaged; results were variable but, on average, the improvement in strength exceeded 20% in the quadriceps and shoulders. Even though the acquisition of functional abilities was short-lived, all the patients were pleased, and two of them wished to pursue IVIG treatments independently. It is not surprising that acute clinical improvements were not accompanied by histological changes, since morphological changes should require long-term treatments and, perhaps, muscle regeneration.

There are several possible explanations for the beneficial clinical effects of IVIG despite the absence of evidence for increased glycoprotein sialylation. The salutary responses to IVIG could have been due to its anti-inflammatory actions [47,48]; patient 3 had previous evidence of central nervous system inflammation and patient 4 had a muscle biopsy read as possible polymyositis. Nevertheless, the myopathy in HIBM is not considered to be inflammatory, and muscle biopsies performed at the NIH on all four patients lacked signs of inflammation. A second possibility is that minor increases in sialylated muscle glycoproteins, responsible for improved muscle function, were present transiently after IVIG treatment, but were undetectable in our assay. A third possibility is that the muscle we biopsied, the quadriceps, had minimal baseline reduction in glycoprotein sialylation so that any increased sialylation after therapy could not be appreciated. Finally, the beneficial clinical effects might not be at all related to sialylation.

Ours was clearly a pilot study, clarifying some issues for future therapeutic investigations. For example, we documented that muscle strength testing provides an objective outcome parameter, with fixed dynamometry detecting small and large changes in strength, complemented by more subjective measures of muscle function [49]. We also determined that muscle histology, immunohistology and sialylated glycoprotein quantitation by immunoblotting do not provide reliable measures of improvement, at least in the short term. These parameters require further investigation using longer treatment regimens and greater amounts of sialic acid supplementation. One possibility would be to administer only the Fc portion of immune globulin, which contains the bulk of the sialic acid residues and has ten times the anti-inflammatory efficacy of the complete molecule [47]. Even so, IVIG is not likely to be the treatment of choice for HIBM, because it must be administered repeatedly, its mode of delivery requires hospitalization, its cost is considerable, and it has side effects. However, the finding of definitive improvements, albeit moderate and transient, attributable to IVIG suggests that the provision of sialic acid holds therapeutic promise.

Our next pursuit will involve administration of ManNAc, an uncharged sugar situated in the sialic acid synthetic pathway after the rate-limiting UDP-GlcNAc 2-epimerase step. Residual ManNAc kinase activity in HIBM patients, or ancillary kinases such as GlcNAc kinase [50], might convert ManNAc into ManNAc-6P for subsequent synthesis of sialic acid. In fact, hyposialylated, *GNE*-deficient mouse embryonic stem cells became resialylated after their growth medium was supplemented with ManNAc [51]. Furthermore, ManNAc supplementation has resulted in prolonged survival of a mouse model of HIBM [52], and could yield increased glycoprotein sialylation in the tissues of humans with HIBM.

## Conclusion

Our findings indicate that IVIG treatment has a salutary clinical effect on the myopathy of HIBM. Since HIBM results from defective sialic acid biosynthesis and is not associated with inflammation, the benefits of IVIG treatment could be related to the high sialic acid content of IgG. These results prompt consideration of other sources of sialic acid as therapy for HIBM, and identify suitable outcome parameters for future clinical trials.

## Abbreviations

HIBM, Hereditary Inclusion Body Myopathy; UDP-GlcNAc, uridine diphosphate-N-acetylglucosamine; ManNAc, N-acetylmannosamine; IVIG, intravenous immune globulin G, NCAM, neural cell adhesion molecule; IEF, isoelectric focusing

## Competing interests

The author(s) declare that they have no competing interests.

## Authors' contributions

SS contributed to conception and design, clinical organization and data collection, and drafting of the manuscript; GR collected the muscle samples and performed immunohistochemistry; MH-L designed the quantitative muscle strength protocol; GJ and JS performed quantitative muscle strength and functional testing; IM and CC performed biochemical analyses including isoelectric focusing and immunoblotting; BS performed swallowing evaluations and tongue strength measurements; HD provided imaging support for immunohistochemistry and preparation of figures; DK contributed to conception and design; MH contributed to conception and design, supervised laboratory analysis and interpretations, and drafting the manuscript; MCD contributed to conception and design and provided clinical expertise on IVIG treatment and muscle histology; WAG substantially contributed to trial conception and design, drafting of the clinical protocol for IRB approval, and drafting the manuscript. All authors read and approved the final manuscript.

## Pre-publication history

The pre-publication history for this paper can be accessed here:



## Supplementary Material

Additional file 1 **Isoelectric focusing of serum transferrin and Apo C-III**. **A**. Normal sialylation of transferrin (N-linked glycoprotein) in all four patients before IVIG (Pre), after IVIG loading (Mid), and after the treatment period (Post). Samples of patients 3 and 4 were electrophoresed on a gel different from the gel for samples 1 and 2. Transferrin sialo-isoforms are indicated by their charge (2–6). **B**. Normal sialylation of Apo C-III in all four patients before IVIG (Pre), after IVIG loading (Mid), and after the treatment period (Post). Normal control sera (NC_1 _and NC_2_) exhibit three bands; treatment with sialidase (S) reduces this to one main band and a minor band. Samples of patients 3 and 4 were electrophoresed on a gel different from the gel for samples 1 and 2. **Apo C-III**_0 _= asialo-; **Apo C-III**_1 _= monosialo-; **Apo C-III**_2 _= disialo-Apo C-III isoforms. Click here for file

## Figures and Tables

**Table 1:** Laboratory values at baseline, after loading with IVIG (1 g/kg × 2), and at the end of IVIG treatment.

	**Baseline**				**Post-load**				**End of study**				**Normal range**
**Patient**	**1**	**2**	**3**	**4**	**1**	**2**	**3**	**4**	**1**	**2**	**3**	**4**	
**Hemoglobin (g/dL)**	13.1	14.4	13.2	14.3	11.2	13.8	11.2	13	12.1	13.8	12.3	11.4	11.1–16.7
**WBC (K/uL)**	8.94	7.57	7.39	7.28	6.82	6.11	8.06	**2.99**	6.94	6.16	6.34	4.85	3.3–9.6
**Platelets (K/uL)**	282	230	284	225	226	209	262	207	267	222	256	220	154–380
**ESR (mm/h)**	17	7	23	14	39	38	**96**	**86**	30	20	**57**	**73**	0–42
**Creatinine (mg/dL)**	0.3	0.7	0.5	0.4	0.4	0.8	0.6	0.6	0.3	0.7	0.6	0.5	0.7–1.4
**BUN (mg/dL)**	15	14	12	13	12	**7**	8	8	16	15	13	17	8–22
**ALT (U/L)**	**42**	36	18	36	31	37	16	30	**60**	**81**	18	**62**	6–41
**AST (U/L)**	30	31	22	23	21	31	15	23	**36**	**68**	31	**52**	9–34
**CPK (U/L)**	320	**475**	98	130	244	**451**	50	87	229	**629**	78	94	38–386
**Cholesterol (mg/dL)**	195	**203**	195	**207**	133	153	144	156	168	179	175	170	100–200
**Total protein (g/dL)**	6.4	6.1	6.1	6.3	**7.8**	**8.7**	**9.5**	**9.1**	7.1	7.2	7.3	7	6.0–7.6
**Albumin (g/dL)**	3.7	3.7	**3.2**	**3.5**	**2.9**	**3.4**	**2.6**	**3**	**3.3**	**3.5**	**2.8**	**3.1**	3.7–4.7
**IgG (mg/dL)**	1280	1180	955	997	**3550**	**4280**	**5670**	**4440**	**2150**	**2350**	**2340**	**2310**	642–1730
**Urine protein (mg/d)**	150	92	**294**	97	<30	<30	<30	<30	79	95	**364**	142	30–150
**Urine glucose (g/d)**	0.07	0.07	0.07	0.09	**14.4**	**30.8**	**6.4**	**12.2**	0.07	0.07	0.10	0.08	0–0.49

Abnormal values are in bold. Normal range encompasses values for males and females.

Baseline values are average of results on two different days.

**Table 2:** Strength of muscle groups of 4 HIBM patients at baseline and after IVIG treatment.

	**Hip Flexion**	**Ankle Dorsiflexion**	**Elbow**		**Wrist**		**Hand**	
			
			**Flexion**	**Extension**	**Flexion**	**Extension**	**Grip**	**Pinch**
**Baseline**^**a,b**^	3.21 ± 0.84	3.50 ± 2.15	4.71 ± 1.42	3.36 ± 1.09	5.24 ± 0.86	4.55 ± 0.46	7.43 ± 1.63	1.02 ± 0.22
**Post-Load**^**b**^	3.93 ± 0.74 (+22%)	2.53 ± 1.55 (-28%)	5.56 ± 1.60 (+18%)	3.30 + 0.98 (-2%)	5.81 ± 1.07 (+11%)	5.15 ± 0.58 (+13%)	7.85 ± 1.65 (+6%)	1.03 ± 0.16 (+1%)
**End of study**^**b**^	3.63 ± 0.62 (+13%)	4.28 ± 2.87 (+22%)	5.45 ± 1.55 (+16%)	3.36 ± 1.03 (0%)	6.25 ± 1.15 (+19%)	5.78 ± 0.93 (+27%)	7.83 ± 1.59 (+5%)	1.37 ± 0.25 (+35%)

^a^Values are given in kg as means ± SEM. Percent changes are in parentheses. Values for both the right and left sides were included in the analyses.

^b^Baseline: Before treatment; Post-Load: Day 1 after IVIG load (1 g/kg × 2); End of study: 4 weeks after the initial IVIG load.
