# Supplementary material for: Intravenous immune globulin in hereditary inclusion body myopathy: a pilot study
Source: BMC Neurol. 2007 Jan 29;7:3. doi: 10.1186/1471-2377-7-3 (PMC1790898; doi:10.1186/1471-2377-7-3)
Supplement: Additional file 1 — Isoelectric focusing of serum transferrin and Apo C-III. A. Normal sialylation of transferrin (N-linked glycoprotein) in all four patients before IVIG (Pre), after IVIG loading (Mid), and after the treatment period (Post). Samples of patients 3 and 4 were electrophoresed on a gel different from the gel for samples 1 and 2. Transferrin sialo-isoforms are indicated by their charge (2–6). B. Normal sialylation of Apo C-III in all four patients before IVIG (Pre), after IVIG loading (Mid), and after the treatment period (Post). Normal control sera (NC1 and NC2) exhibit three bands; treatment with sialidase (S) reduces this to one main band and a minor band. Samples of patients 3 and 4 were electrophoresed on a gel different from the gel for samples 1 and 2. Apo C-III0 = asialo-; Apo C-III1 = monosialo-; Apo C-III2 = disialo-Apo C-III isoforms. [file 1471-2377-7-3-S1.ppt]

## Slide 1
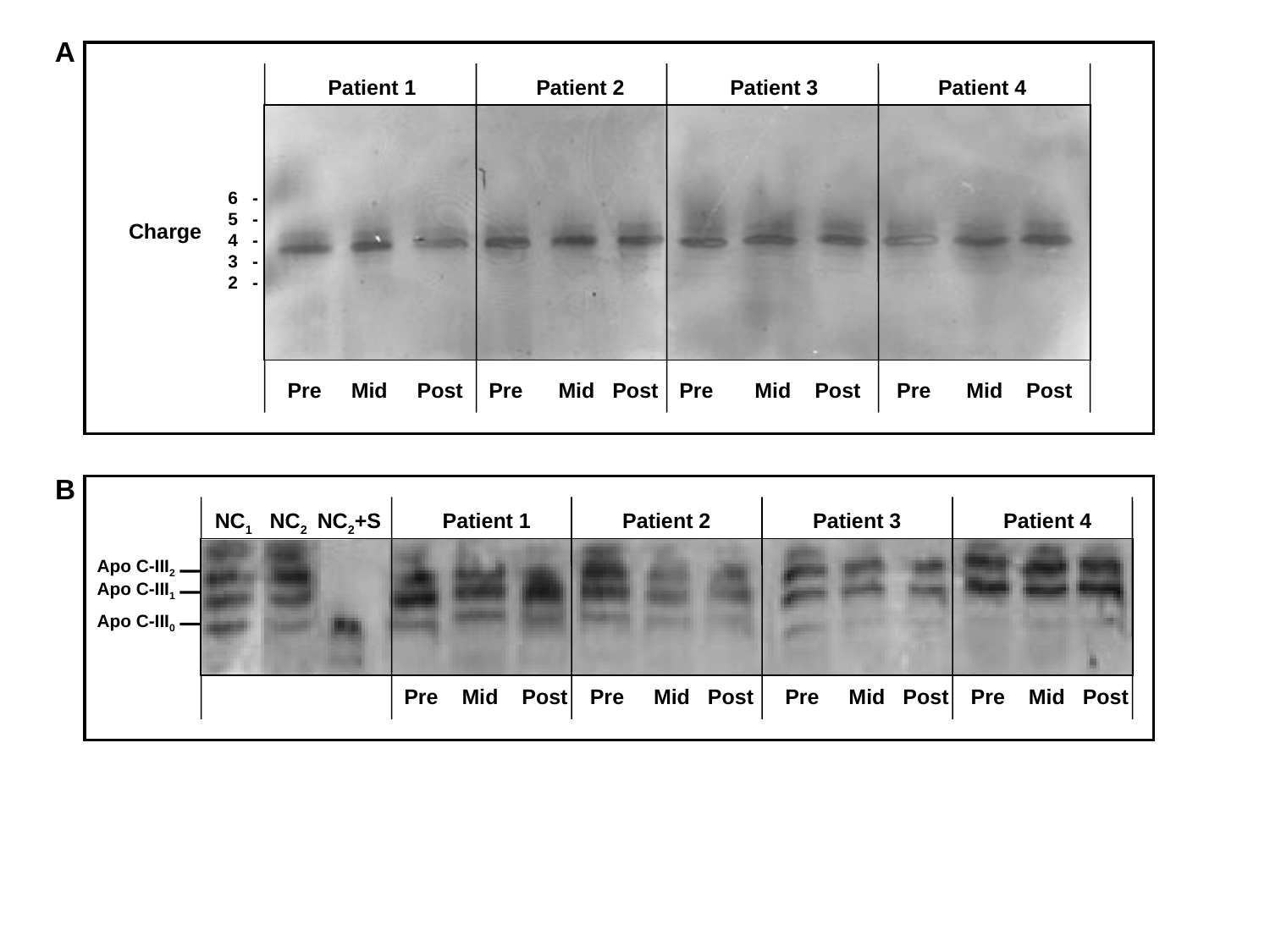

A
Patient 1
Patient 2
Patient 3
Patient 4
 6 -
 5 -
Charge
 4 -
 3 -
 2 -
Pre Mid Post
Pre Mid Post
Pre Mid Post
 Pre Mid Post
B
 NC1 NC2 NC2+S
Patient 1
Patient 2
Patient 3
Patient 4
Apo C-III2
Apo C-III1
Apo C-III0
Pre Mid Post
 Pre Mid Post
Pre Mid Post
 Pre Mid Post
